# Book Reviews

**Published:** 1895-05

**Authors:** 


### *BOOK REVIEWS.*

THE STANDARD DICTIONARY OF THE ENGLISH LANGUAGE. By Isaac K. Funk, D.D., Editor in Chief, Francis A. March, LL.D., L.H.D., Consulting Editor, and Daniel S. Gregory, D.D., Managing Editor. Associate Editors: John D. Champlin, M.A.; Arthur E. Bostwick, Ph.D., and Rossiter Johnson, Ph.D., LL.D. Sold only by subscription. Published by Funk & Wagnalls Company, 30 Lafayette Place, New York.

In bringing forward this immense work the editors and publishers have made the English-speaking world their debtors. Nothing has been spared, neither time, nor labor, nor expense, to make this indeed the standard dictionary of the English language. For nearly five years about 250 editors and authorites in special departments of knowledge have been engaged upon the definitions, and 500 special readers have been looking up appropriate quotations from the best English writers. The work contains 2,300 pages, and 300,000 vocabulary terms, which is 75,000 more than any other dictionary of the language.

Among the distinguishing features of the Standard should be mentioned: (1) The most common meaning of a word is placed first; the order of usage being observed, rather than the historical order; (2) The adoption of the *scientific alphabet,* as recommended by the American Philological Association, in indicating the pronunciation of words; (3) Obsolete and dialectic words are placed in the appendix; (4) Antonyms, as well as synomyms, are given where this is thought proper; (5) In the vocabulary only proper names are printed with initial capital letters; (6) All words relating to any particular science have been referred for definition to persons specially learned in that science. The Standard is the first dictionary to make use of the "scientific alphabet," and while the indicated pronunciations and diacritical marks may not be very clear at first, doubtless they will prevail in the end. One has only to look for new words to see how completely the Standard has been brought up to date. Electricity has probably contributed more new words to the language in late years than any other branch of science. All of these are here, and fully defined. Among other new words, now included for the first time in a general dictionary we notice appendicitis, criminology, electricute, kodak, mafia, populist, pharmacal, volar, jiggered, fin-de-siècle, delicatessen, snolly-goster, gutter-snipe, cable-car, etc. Regular medicine is represented on the editorial staff by Dr. Frank Baker, of Washington, and Dr. Frank P. Foster and Dr. T. M. Prudden, of New York, the latter having charge of words relating to bacteriology. Homeopathic and eclectic medicine are also represented by good authorities. From a medical point of view the Standard is infinitely ahead of any other general dictionary that we know of, and we believe that the same may be said, *mutatis mutandis*, for other departments of science. It is the newest, most convenient and most complete dictionary of the language, and stands for the combined literary and scientific scholarship of the present day. It is indeed an "intellectual collaboration," the highest intelligence of the English-speaking world having been laid under tribute in its production. We quite indorse what one critic has already said of it, that "it passes the wit of man to suggest anything which ought to have been done that has not been done to make the dictionary a success."

---

TRANSACTIONS OF THE ANTISEPTIC CLUB. Reported by Albert Abrams, Member of the San Francisco Medical Profession. Illustrated. E. B. Treat, 5 Cooper Union, New York.

This is about the poorest excuse for a book that we have seen in many a day. The only explanation for its existence which we can offer is that the author is a victim of the complaint, common among medical men, known as *cacoethes scribendi*. If the learned members of the Antiseptic Club wished to have their transactions put into print they certainly made a most woeful mistake in their editor. The few examples of good wit in the book are aged, and we must respect them on that account; they are borrowed, and generally without credit. The numerous original and abortive attempts at wit, found on every page, are of a low order, aimed usually at the medical profession, and suggest the thought that the author must have once served a term as end man in a minstrel show. One can imagine how a very readable book could be made from such a subject. As an example of how *not to do it* the volume before us is a dazzling success.

---

ANTISEPSIS AND ANTISEPTICS. By Charles M. Buchanan, M. D., Professor of Chemistry and Toxicology, National University, Washington, D. C. Published by the Terhune Company, Newark, N. J.

This will be found a very convenient little work on the subject of which it treats. The first forty-six pages enter very fully into the history and development of the germ theory from the earliest times to the advent of Lister and the present day. The next chapters discuss the products of cellular and bacterial activity, immunity and infection, etc. The last chapters relate to antiseptics proper, describing their relative value, methods of application, their use in medicine, surgery, obstetrics and gynecology, etc.

---

THE INTERNATIONAL MEDICAL ANNUAL AND PRACTITIONERS' INDEX: A Work of Reference for Medical Practitioners. Thirteenth year. New York. E. B. Treat, 5 Cooper Union. Price, $2.75.

This book hardly needs a review as it has been before the public for thirteen years. It recommends itself by keeping up with the latest methods of treating diseases. Also the latest discoveries in medical and surgical science. The treatment of diphtheria by antitoxine, by Dr. Armand Ruffer, is without doubt the most important advance during the year. The article was too late for classification and will be found on page 525.

This volume of the Annual is in thorough keeping with its predecessors, and we heartily recommend it.

